# Intensity Variation Normalization for Finger Vein Recognition Using Guided Filter Based Singe Scale Retinex

**DOI:** 10.3390/s150717089

**Published:** 2015-07-14

**Authors:** Shan Juan Xie, Yu Lu, Sook Yoon, Jucheng Yang, Dong Sun Park

**Affiliations:** 1 Institute of Remote Sensing and Earth Science, College of Science, Hangzhou Normal University, Hangzhou 311121, China; E-Mail: shanj_x@hotmail.com; 2 Division of Electronic and Information Engineering, Chonbuk National University, Jeonju561-756, Korea; E-Mail: dspark@jbnu.ac.kr; 3 Department of Multimedia Engineering, Mokpo National University, Jeonnam534-729, Korea; E-Mail: syoon@mokpo.ac.kr; 4 College of Computer Science and Information Engineering, Tianjin University of Science and Technology, Tianjin 300222, China; E-Mail: jcyang@tust.edu.cn

**Keywords:** intensity variation, finger vein recognition, image enhancement, guided filter, single scale retinex

## Abstract

Finger vein recognition has been considered one of the most promising biometrics for personal authentication. However, the capacities and percentages of finger tissues (e.g., bone, muscle, ligament, water, fat, *etc.*) vary person by person. This usually causes poor quality of finger vein images, therefore degrading the performance of finger vein recognition systems (FVRSs). In this paper, the intrinsic factors of finger tissue causing poor quality of finger vein images are analyzed, and an intensity variation (IV) normalization method using guided filter based single scale retinex (GFSSR) is proposed for finger vein image enhancement. The experimental results on two public datasets demonstrate the effectiveness of the proposed method in enhancing the image quality and finger vein recognition accuracy.

## 1. Introduction

As a newly emerging biometric, finger vein recognition has attracted significant attention and achieved remarkable development during the last decade. Compared with other traditional biometrics, the FVRS has the advantages of low cost, easy collection of images with contactless operation, liveness, and, respectively, smaller size of the imaging device [1,2,3,4,5,6,7,8].

Finger veins are subcutaneous structures that randomly develop inside a finger. They are viewable with reflected light due to the peak absorption of near infrared illumination by oxygenated and de-oxygenated hemoglobin in the blood [9]. This makes finger vein media resistant to theft and forgery. In practice, however, FVRSs suffer from external factors such as imaging models [10] and uneven illumination [11,12], and internal factors including scattering [13] and finger tissue [14]. These factors cause the finger vein images to become unstable and have low contrast. Thus, it is difficult for FVRSs to achieve reliable and accurate recognition performance in real scenarios.

To address this problem, finger vein image enhancement has widely been researched over the past few years to enhance the quality of finger vein images. Pi *et al.* [14] combined an edge-preserving filter, an elliptic high-pass filter, and histogram equalization to enhance the contrast of finger vein images. An illumination component was generated by the convolution of the original image with its filtered images using the average filter in [12] to alleviate the effect of uneven illumination for finger vein authentication. Considering the variations of vein-coursing directions, Yang *et al.* proposed the utilization of different oriented filters to allow enhancement of the finger vein image [15,16]. Park *et al.* [17] proposed enhancing the image quality using direction and thickness of the vein lines for finger vein recognition. Yang *et al.* [13] proposed a biological optical model for estimation and removal of the light scattering component. Lee *et al.* [18] introduced a finger vein image restoration method to deal with skin scattering and optical blurring. These methods can, respectively, enhance finger vein images to some extent; however, little attention has been given to the factor from finger tissue, which also results in poor quality of the finger vein image.

The tissue components of a finger, including fat, bone, skin, muscle, water, *etc.*, are the same for each individual. However, the capacities and percentages of these tissue components vary among a person, which is easily proven by the variation in thickness of fingers. For some finger vein images, the shape of blood vessels is projected poorly due to the effect of interruption caused by the components inside the finger. Thus, the contrast between the venous and non-venous regions of the images is poor. It was mentioned by Pi *et al.* [14] that tissue structure in different parts of the finger could result in low quality finger vein images. However, they did not propose a special method to cope with the finger tissue to allow better finger vein recognition. Yang *et al.* [19] proposed a finger-image restoration method considering skin layer structure, where a Gaussian-point spread function (PSF) model and two depth-PSF models were adopted. However, there are no reports about enhancement accuracy through use of the methods.

In the present paper, the factor that results in poor finger vein images due to the finger tissues is named intensity variation. Even though imaging devices are relatively less sensitive to the external factor, this factor is still not avoidable, since each finger can make a different intensity variation according to its tissue structure. Therefore, an additional operation is required to eliminate the intensity variation in finger vein images. To this end, we propose an intensity variation normalization method using guided filter based single scale retinex (GFSSR). Inspired by the assumptions in the single scale retinex (SSR) algorithm, a finger vein image is assumed to be regarded as the multiplication of its intensity variation and the reflectance images. The original multiplication relation between the intensity variation and reflectance images can be translated to a subtraction operation with the use of a logarithm operation. To obtain an accurate intensity variation image, the guided filter [20] is adopted in SSR to smooth an input image due to its adjustable edge-preserving smoothing ability. The experimental results obtained for the public datasets MMCBNU_6000 [21] and UTFVP [22] demonstrate that the proposed method can effectively alleviate the intensity variation in finger vein images and enhance the image quality, thereby improving the matching performance.

The reminder of this paper is organized as follows: Section 2 introduces the intensity variation in detail. The proposed method using guided filter for intensity variation normalization is reported in Section 3. Section 4 reports the experimental results. Finally, the conclusion and suggestions for future work are given in Section 5.

## 2. Intensity Variation

**Figure 1:**
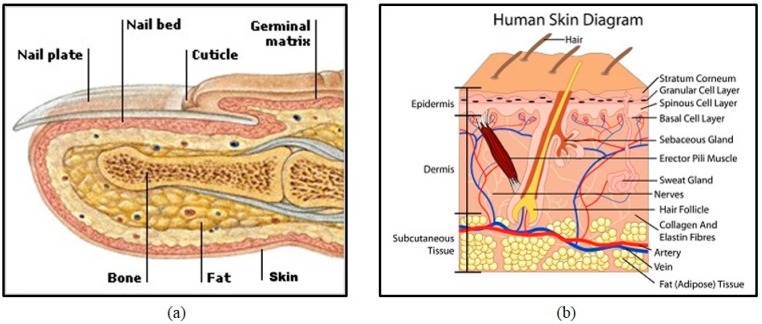
Human finger structure: (**a**) cross-section [23] and (**b**) skin diagram [24].

As shown in Figure 1, a finger usually contains fat, bone, skin, and nail components. Veins are located in the subcutaneous layer deep in the skin with fat, connective tissue and other tissues. All the tissues and organs inside a finger can absorb near infrared (NIR) illumination with different absorptivity. As oxyhemoglobin and deoxyhemoglobin in blood vessels absorb more NIR radiation than the other substances, vein vessels are shown in darker color while the other tissues are presented with a brighter background in the captured vein image.

As mentioned above, while each individual has the same types of finger tissues, the capacities and percentages of each tissue vary from person to person. Thick fingers contain more fat, while thin fingers contain less fat. The captured images from a thin finger usually have higher image brightness than those from a thick finger. The acquired finger vein images from different individuals show different global and local contrast, especially between the venous and non-venous regions.

**Figure 2:**
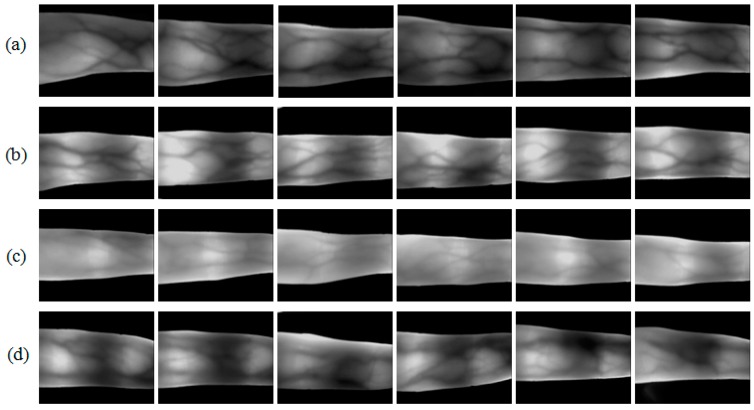
Image samples from our available database MMCBNU_6000 [21]: (**a**–**d**) are finger vein image samples collected from four volunteers P1–P4, respectively. Each row shows six images from six different fingers of one individual.

Figure 2 shows four groups of finger vein images coming from MMCBNU_6000. Each row depicts six images taken from six fingers of a volunteer. The figure clearly illustrates that the images from different individuals displayed different finger structures and thicknesses. In addition, there were various image contrasts for each individual due to intensity variation. The first individual (P1) had the thickest fingers. Images (Figure 2a) collected from P1 showed good image contrast between the venous and non-venous areas. The individuals of P2 and P3 have thinner fingers than P1; therefore, the volume of each tissue was larger in P1 than those in P2 and P3. Thus, Figure 2b,c displayed higher brightness on the whole, as compared with the image brightness shown in Figure 2a. Furthermore, the images captured from P2 and P3 displayed different global and local image contrast due to the effect of intensity variation. Since the bone in the finger joint is articular cartilage and can easily be penetrated by infrared light [15], the joint part in the image is always shown in brighter gray values. This resulted in brighter local areas in each of the captured images shown in Figure 2. However, due to intensity variation, the local image contrast in the finger joint parts was much more obvious in Figure 2b than those in other images. Images displayed in Figure 2c showed lower global image contrast than those in the other images. Figure 2d shows good global image contrast; the regions between the venous and non-venous areas were not clear. This may have resulted from the presence of thick fat or muscle near the finger veins. Although the thickness of fingers from individual P2 and P4 are almost same, the image contrasts are different due to the intensity variation.

Intensity variation in finger vein recognition is an internal factor that results in poor quality of the finger vein images and is inevitably generated in the process of imaging. No matter what kind of imaging model or device is used, intensity variation appears in the finger vein images, degrades the image contrast and thereby degrading the matching performance of an FVRS. Thus, a specialized method that focuses on alleviating the effect of intensity variation would be beneficial for enhancing the quality of finger vein images and the matching performance of the FVRS.

## 3. Proposed Intensity Variation Normalization Method

In this section, the guided filter and single scale retinex algorithm are first reviewed, after which the proposed guided filter based single scale retinex method is described in detail for normalization of intensity variation in finger vein recognition.

### 3.1. Guided Filter

Guided filter [20] is an effective smoothing filter and its edge-preserving smoothing ability can be controlled by parameters. Taking into account this property and vague edges in some finger vein images due to intensity variation, guided filter is adopted in the present paper for smoothing the input images. The key assumption of the guided filter is a local linear model between the guidance *S*, and the filter output *g*. The guidance image is guided for smoothing an input image. It is supposed that *g* is a linear transform of *S* in a window *w_k_*, centered at pixel *k* [20]:

(1) $$g_{i}=a_{k}S_{i}+b_{k}\;\;\forall i\in w_{k}$$

where $\left(a_{k},b_{k}\right)$ are some linear coefficients assumed to be constant in *w_k_*. This local linear model ensures that *g* has an edge only if *S* has an edge since ∇g=a∇S.

To determine the linear coefficients, the cost function that minimizes the difference between the input image *L* and the output is as follows.

(2) $$E(a_{k},b_{k})=\sum_{i\in w_{k}}\left({\left(a_{k}S_{i}+b_{k}-L_{i}\right)}^{2}-\varepsilon a_{k}^{2}\right)$$

The solution of Equation (2) is given by the linear regression method:

(3) $$a_{k}=\frac{\frac{1}{\left|w\right|}\sum_{i\in w_{k}}S_{i}L_{i}-\mu_{k}{\bar{L}}_{k}}{\sigma_{k}^{2}+\varepsilon }$$

(4) $$b_{k}={\bar{L}}_{k}-a_{k}\mu_{k}$$

where ε is a regularization parameter preventing $a_{k}$ from being too large, μ and $\sigma_{k}^{2}$ are the mean and variance of *S* in $w_{k}$, and ${\bar{L}}_{k}$ is the mean of *L* in $w_{k}$. |w| is the number of pixels in $w_{k}$. Hence, after computing $\left(a_{k},b_{k}\right)$ for all patches $w_{k}$ in the image, the filter output can be computed by:

(5) $$g_{i}=\frac{1}{\left|w\right|}\sum_{k:i\in w_{k}}\left(a_{k}S_{i}+b_{k}\right)={\bar{a}}_{i}S_{i}+{\bar{b}}_{i}$$

where ${\bar{a}}_{i}$ and ${\bar{b}}_{i}$ are the mean values in $a_{k}$ and $b_{k}$, respectively.

Due to the linear model between the guidance and the filter output, the guided filter has a better edge-preserving smoothing property than other filters. The non-approximate manner in implementation results in good quality of the generated results. Furthermore, the linear running time of the algorithm depends only on the number of pixels in the image.

### 3.2. Single Scale Retinex Algorithm

Single scale retinex (SSR) [25] is based on the assumption that an observed image *L* can be regarded as the multiplication of the illumination *I* and the reflectance images *R*. *R* can be considered as the textures without any illumination variations. Moreover, it is assumed that the reflectance changes sharply and that illumination changes smoothly. There are a lot of methods for decomposition of the intensity into these two components and the SSR algorithm is used as a technique to enhance images in various applications [26]. The mathematic description for each pixel (x,y) in an image is defined as follow:

(6) L(x,y)=I(x,y)×R(x,y)

To eliminate the illumination from the captured image, a subtraction operator is applied in the logarithm domain.

(7) logR(x,y)=logL(x,y)−logI(x,y)

Since SSR is based on the idea that the illumination component tends to change smoothly, contrary to the reflectance, the illumination image *I* can be estimated by the convolution operation of the Gaussian filter on the captured image *L*. The operation for each pixel (x,y) is as shown below:

(8) I(x,y)=L(x,y)×F(x,y)

(9) $$F(x,y)=\frac{1}{2\pi \sigma^{2}}e^{-\frac{x^{2}+y^{2}}{2\sigma^{2}}}$$

Substituting Equation (8) into Equation (7), we have

(10) logR(x,y)=logL(x,y)−log(L(x,y)×F(x,y))

**Figure 3:**
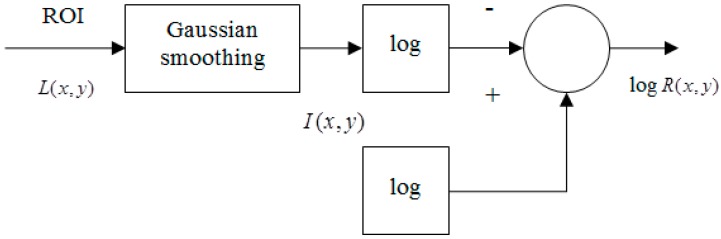
Block diagram of the SSR algorithm.

Consequently, log R(x,y) is the retinex output, called single scale retinex (SSR), while it is also the illumination-normalized output. The block diagram for SSR is shown in Figure 3.

### 3.3. Proposed GFSSR

Each tissue in a finger can absorb NIR illumination to different extents, causing undesired intensity variations in a finger vein image. The intensity variation normalization proposed in this paper is designed to eliminate this effect. Inspired by the assumption of SSR, it was assumed that a captured finger vein image *L* could be regarded as the multiplication of the intensity variation *IV* and the reflectance *R*. The mathematic description for each pixel (x,y) can be represented as follow:

(11) L(x,y)=IV(x,y)×R(x,y)

As mentioned in Section 3.2, it is common that an intensity variation image *IV* can be estimated by the convolution operation of the smoothing filter on the captured image *L*. Here, we use a guided filter as a smoothing filter to obtain an intensity variation image of a finger vein image.

**Figure 4:**
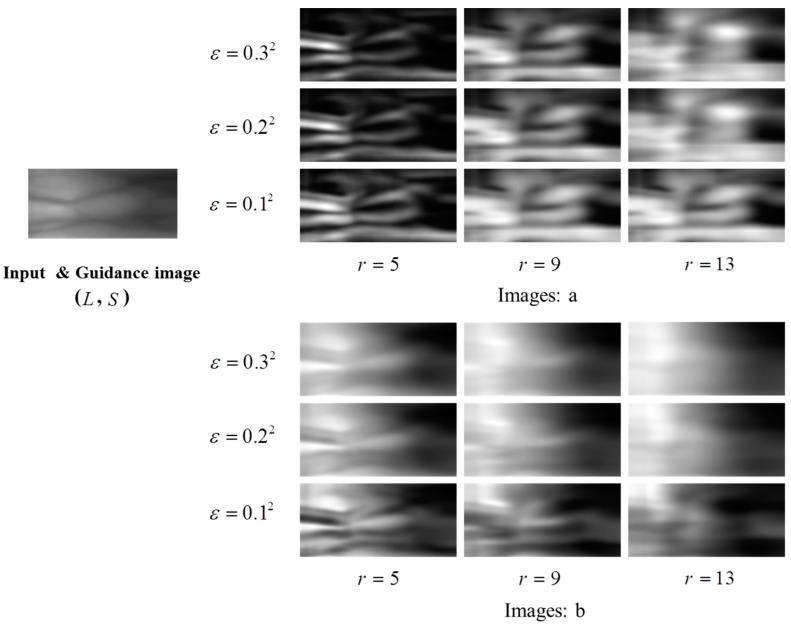
Examples of parameters (*a*,*b*) of the guided filter according to different parameters (ε,*r*).

A result of guided filter at a point (x,y), is obtained by a weighted sum of an intensity value at (x,y) in a given guidance image, *s*, and an average of a patch centered at (x,y) in a given input image, μ(x,y). *a* and *b* are used as its weights, which are determined by whether a patch centered at a point in the guidance image has relatively high variance, as defined in Equations (3) and (4). If a patch has relatively high variance, *a* becomes relatively large and contributes to g(x,y) more than μ(x,y). Otherwise, μ(x,y) contributes to g(x,y) more than s(x,y). As shown in Figure 4, *r* is a radius of a patch and becomes larger. A region with relatively high variance also becomes larger and their variance values become smaller, so the contribution of s(x,y) to g(x,y) at a position (x,y) becomes smaller than in a case of using a smaller *r*. While its smoothing effect becomes stronger, its edge-preserving effect becomes weaker, but its tendency still remains. Therefore, the guided filter can work pursuing for a given purpose, when its parameter values and a guidance image are chosen properly.

**Figure 5:**
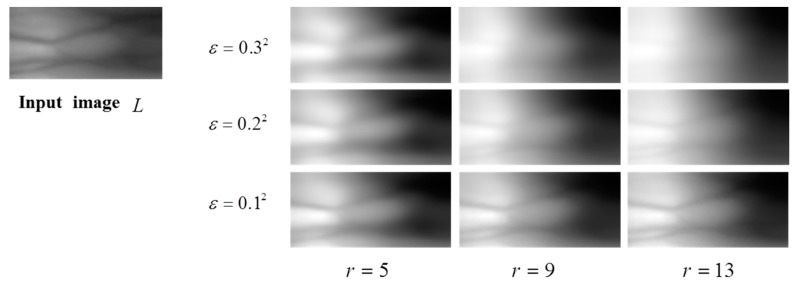
Outputs of guided filter with different parameters (ε,*r*). The guidance image is identical to the input image.

**Figure 6:**
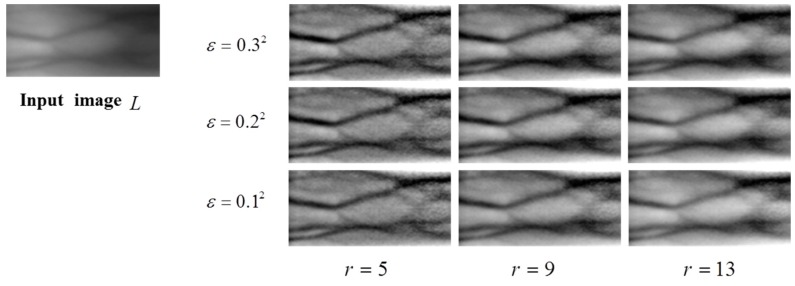
Outputs of IV normalization using GFSSR with the same parameters (ε,*r*) as those in Figure 5.

Finger vein images include mostly background but sparsely curves, which may be blurred by any external factor. Through a guided filter, most background regions can be smoothed in their local means, since their variances are relatively small. Meanwhile, since vein curves are covered in region with relatively high variances, their curves also remain as a filtering result, but their intensity become lower due to larger *r*. Therefore, as shown in Figure 5, when we look at a result of a finger vein image filtered by a guided filter, we can see that it includes most of background smoothed by local means and weak vein curves. When this result is subtracted from its original image by GFSSR process, it contributes on not only removing some intensity variation with smoothing effects but also on reducing blurring effects of curves, as shown in Figure 6.

**Figure 7:**
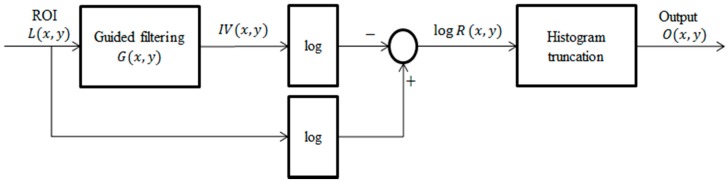
Block diagram of the proposed GFSSR algorithm.

The proposed Guided filter based SSR (GFSSR) algorithm can be described as:

(12) logR(x,y)=logL(x,y)−log(L(x,y)×G(x,y))

where IV(x,y) is estimated by the convolution operation of the guided filter, G(x,y), on the captured image *L*, IV(x,y)=L(x,y)×G(x,y). The block diagram of GFSSR is depicted in Figure 7.

In this paper, the proposed GFSSR is performed directly on regions of interest (ROI) in the images. To accurately localize ROI from the captured images, the robust finger vein ROI localization method proposed in our previous research [27] is adopted. The guidance image is selected as the input image. Compared with the input images, the enhanced images after intensity variation normalization using the proposed method have higher image contrast and quality, as shown in Figure 6. The use of guided filter in the proposed method could not only smooth the captured image well but also cause loss of some vein information after the subtraction operation. To address this problem, histogram truncation is adopted to compensate for the loss of the vein information in this process, for further enhancement of the image quality.

## 4. Experimental Results

In order to ascertain the performance improvement using the proposed method, different methods for illumination normalization and image enhancement were implemented for comparison. All the experiments were performed on two public available finger vein database, MMCBNU_6000 [21] and UTFVP [22], using MATLAB (R2013a) on a computer with an Intel Core i3-2120 and 4 GB of RAM.

### 4.1. Dataset

MMCBNU_6000 consists of finger vein images captured from 100 volunteers, coming from 20 different countries. Each subject was asked in the capturing process to provide images of his or her index finger, middle finger, and ring finger of both hands during the capturing process. The collection of each of the 6 fingers was repeated 10 times to obtain 10 finger vein images. Our finger vein database is therefore composed of a total of 6000 images. Each image was stored in “bmp” format at the size of 480 × 640 pixels. The localized ROI image had the pixels size of 64 × 128. Some of the captured image samples and ROI image samples are shown in Figure 2 and Figure 6, respectively.

**Figure 8:**
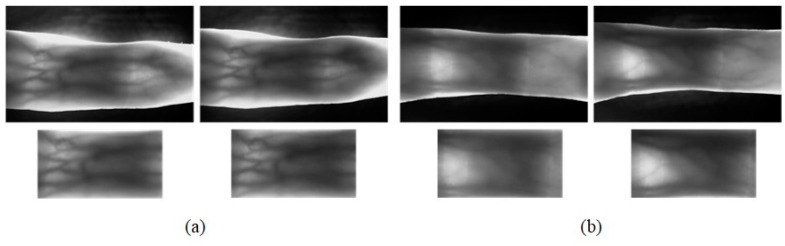
Two groups of images, (**a**) and (**b**), and their ROIs from UTFVP [22].

UTFVP [22] contains 1440 finger vascular pattern images in total which have been collected from 60 volunteer at the University of Twente. Images were captured in two identical sessions with an average time lapse of 15 days. The vascular pattern of the index, ring and middle finger of both hands has been collected twice at each session. Two images were collected for each finger in each session. The captured images have a resolution of 672 × 380. Each image is stored using the lossless 8-bit grey scale PNG format. ROIs of images in UTFVP, extracted using the algorithm proposed in [27], had the resolution of 60 × 120. Some of the finger vascular pattern images from UTFVP and their ROIs are displayed in Figure 8.

### 4.2. Investigation of Optimal Parameters

The performance of FVRS using the proposed GFSSF is dependent on two parameters: the regularization parameter ε and the radius *r* of the window $w_{k}$. In this section, an experiment was designed to explore the optimal parameters using images from MMCBNU_6000. Since the aim of the proposed GFSSF was to enhance the image quality and to improve the matching performance of the FVRS, the equal error rate (EER) was adopted to evaluate the matching accuracies with different parameters. EER is the value whereat the false accept rate (FAR) is equal to the false reject rate (FRR). Discrete wavelet transform (DWT) [27,28] was adopted for feature extraction of the enhanced finger vein images with different combinations of parameters. The nearest neighbor classifier based on cosine distance was employed for matching. In all experiments, each finger was considered as an individual. Five finger vein images from one individual were selected as the training set, while the remaining five images were used as the test set. The number of genuine and imposter matches are 3000(600 × 5) and 1,797,000(600 × 599 × 5), respectively.

**Figure 9:**
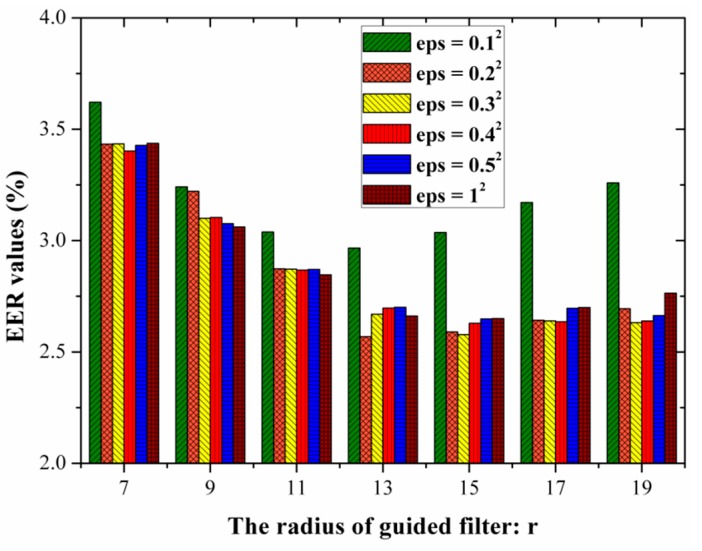
EER values with varying parameters. r∈{7,9,11,13,15,17,19} and $eps=\varepsilon \in \{0.1^{2},0.2^{2},0.3^{2},0.4^{2},0.5^{2},1^{2}\}$.

Figure 9 depicts the EER values with varying parameters. The EER values obtained using DWT for feature extraction directly from the ROI images is 3.93%. It is clearly illustrated in Figure 9 that the proposed GFSSR can enhance the matching performance regardless of the groups of parameters adopted. Furthermore, the performance achieved using the proposed GFSSR with $\left(\varepsilon ,r)=(0.2^{2},13\right)$ is better than those using other groups of parameters. Thus, the optimal values are $\left(\varepsilon ,r)=(0.2^{2},13\right)$, which are adopted in the rest of the experiments on two datasets.

### 4.3. Comparison of Image Enhancement

The experiments carried out in this section aimed to show the effectiveness of the proposed GFSSR for finger vein image enhancement. To this end, several approaches for finger vein image enhancement, including illumination normalization (IN) [12], scattering removal (SR) [13], and other common methods widely used for image enhancement such as histogram equalization (HE), and single scale retinex (SSR) [25] were implemented for comparison. $\sigma^{2}$ is selected as 0.04 in SSR.

**Figure 10:**
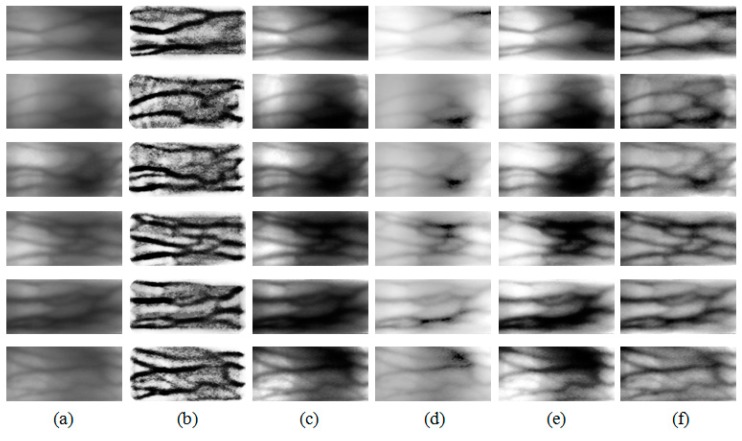
Comparison of finger vein image enhancement using different methods on MMCBNU_6000: (**a**) original ROI images, and enhanced images using (**b**) IN method; (**c**) SR method; (**d**) SSR method; (**e**) HE method; and (**f**) the proposed GFSSR method.

Some ROIs of the samples from MMCBNU_6000 and their enhancements using different methods are displayed in Figure 10. It can be seen that HE can enhance the brightness level. However, as shown in Figure 10e, HE caused level saturation effects in some small regions. This leads to the presence of some dark regions, much darker than those in the input images. The same circumstance appears in the enhanced images using SSR, as shown in Figure 10d. Although the enhanced images using SSR have brighter distribution than the input images, the contrast between venous and non-venous does not show much of an increase. It can be seen in Figure 10b that the images enhanced using IN [12] has good image contrast and clear edges between the venous and non-venous regions. Unfortunately, some of the vein patterns are lost in the local regions. Moreover, the thickness of the veins in Figure 10b increases compared to those in the input images. For images enhanced using SR [13], low image contrast regions in ROI images is still poor in the corresponding regions in Figure 10c. The proposed GFSSR concentrates on investigating the effects of finger tissue on finger vein imaging. Borrowing the adjustable edge-preserving ability of guided filter, the images enhanced using the proposed GFSSR has better image contrast not only in the global image, but also in local regions. In addition, as shown in Figure 10f, the enhanced images have much clearer edges, especially in the vague local regions. Furthermore, the thickness of the veins in Figure 10f remains the same as with those in the input images.

### 4.4. Comparison of Matching Accuracy

Since image enhancement methods for finger vein images aim to enhance the image quality, ultimately to provide improvement of the matching accuracy of the FVRSs, the focus of experiments in this section was on evaluating the improvement of matching performance conferred by each method in Figure 10. In this paper, DWT [12,16], LBP [29], and LPQ [30] are adopted for feature extraction. All the experiments are performed on MMCBNU_6000 and UTFVP. Like the experiment designed in Section 4.2, the EER value was adopted to evaluate the matching accuracies and further to illustrate effectiveness of the image enhancement methods. Cosine distance is used for DWT while Euclidean distance is employed for LBP and LPQ to measure similarity between two images. The nearest neighbor classifier was employed for matching. For MMCBNU_6000 and UTFVP, each finger was considered as an individual.

#### 4.4.1. Experiments Results on MMCBNU_6000

Five finger vein images from one individual in MMCBNU_6000 were selected as the training set, while the remaining five images were used as the test set. The number of genuine and imposter matches are 3000(600 × 5) and 1,797,000(600 × 599 × 5), respectively.

**Figure 11:**
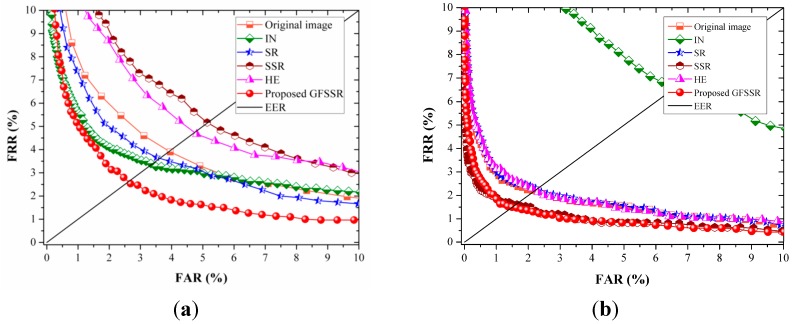

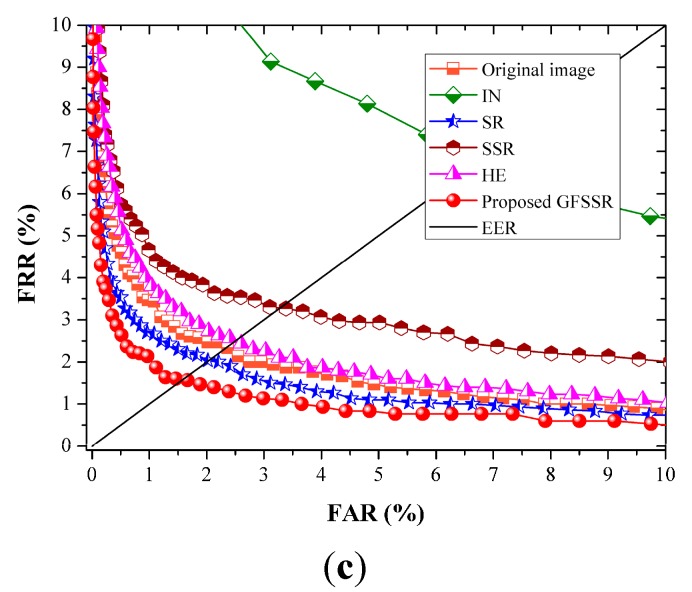
ROC curves of different image enhancement methods on MMCBNU_6000 using different feature extraction algorithms: (**a**) DWT method; (**b**) LBP method; and (**c**) LPQ method.

The receiver operating characteristic (ROC) curves of the different image enhancement methods with different algorithms for feature extraction are depicted in Figure 10. The EER values and the improved ratios calculated based on the EER values obtained using original image are listed in Table 1. The positive ratios represent the corresponding image enhancement algorithm can enhance the matching accuracy while the negative ratios denote the image enhancement method can degrade the matching accuracy. It is clearly shown that SSR, which is effective for facial image illumination normalization, is not beneficial for enhancing the quality of finger vein when using DWT and LPQ for feature extraction. HE exacerbates the poor image contrast in some local regions, as shown in Figure 10e, and thus cannot enhance the matching accuracy. In contrast, SR can enhance the performance a little by using DWT and LPQ for feature extraction. Note that different feature extraction algorithms focus on extracting different kinds of features from an image and different image enhancement methods aims at enhancing images in various styles. Thus, as listed in Table 1, DWT, LBP, and LPQ gave various matching accuracies on different enhanced images. SSR is beneficial for image enhancement when using LBP for feature extraction, but it fails with usage of DWT and LPQ. A similar circumstance appears for images enhanced using IN. These cases demonstrate that IN, SR, SSR and HE are not stable for enhancing image quality. However, it is shown in Figure 11 and Table 1 that the proposed GFSSR has the best performance, no matter which feature extraction was adopted. It results in making lowest EER values, which reduces EER values by 34.6%, 31.8%, and 33.0% compared to the EER values obtained on the original images, using DWT, LBP, and LPQ, respectively.

**Table 1:** EER values and their improved ratio achieved using different feature extraction algorithms on MMCBNU_6000.

Data	EER Values/Improved Ratios		
	DWT [28]	LBP [29]	LPQ [30]
Original image	3.93%	2.20%	2.33%
IN	3.36%/14.5%	6.67%/−203.2%	6.78%/−191.0%
SR	3.71%/5.6%	2.26%/−2.7%	2.03%/12.9%
SSR	5.17%/−31.6%	1.60%/27.3%	3.27%/−40.3%
HE	4.77%/−21.4%	2.18%/1.0%	2.51%/−7.7%
Proposed GFSSR	**2.57%/34.6%**	**1.50%/31.8%**	**1.56%/33.0%**

Taking into account both comparisons of image quality and matching performance, it can clearly be seen that the proposed GFSSR can effectively improve both image quality and the matching performance. Furthermore, the proposed GFSSR takes only 6.3 ms for analysis of an ROI image, which illustrates that it is sufficiently fast in practice.

#### 4.4.2. Experiments Results on UTFVP

**Figure 12:**
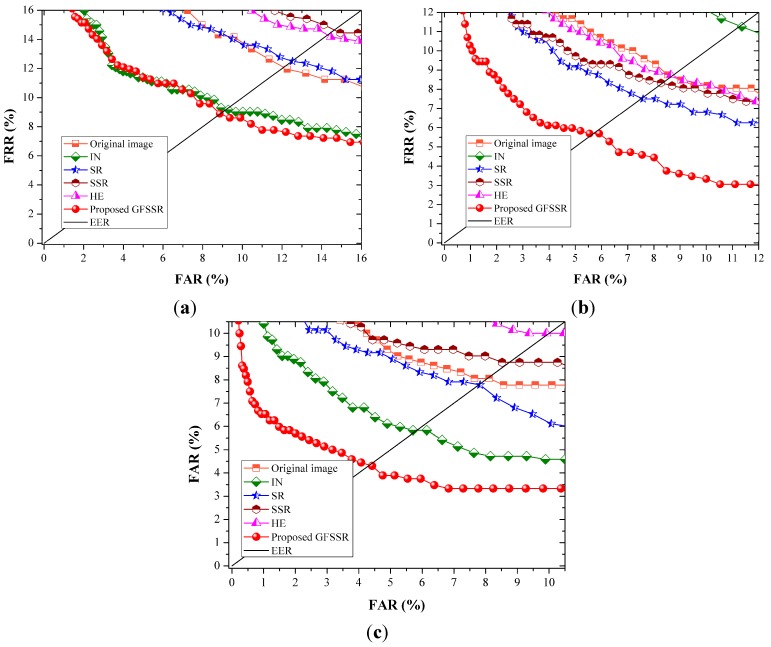
ROC curves of different image enhancement methods on UTFVP using different feature extraction algorithms: (**a**) DWT method; (**b**) LBP method; and (**c**) LPQ method.

For UTFVP, two finger vein images from one individual were selected as the training set, while the remaining two images were used as the test set. The number of genuine and imposter matches are 720(360 × 2) and 258,480(360 × 359 × 2), respectively. Figure 12 shows the ROC curves using DWT, LBP, and LPQ for feature extraction and the corresponding EER values and improved ratios are listed in Table 2.

Compared with the EER values achieved on MMCBNU_6000, the EER values obtained on UTFVP is large, no matter which kinds of feature extraction algorithms are adopted. This may results from the fewer number of images for training than those in MMBNU_6000.However, the proposed GFSSR is stable for image enhancement. It is clearly shown in Table 2 that proposed GFSSR has the best performance, resulting in achieving lowest EER values. It can reduce EER values by 26.6%, 33.9%, and 46.5% using DWT, LBP, and LPQ, respectively. Other image enhancement methods can also reduce EER values to some degrees; however, they are not stable for reducing EER values using all adopted feature extraction methods. IN can enhance matching accuracy when using DWT and LPQ for feature extraction, but it fails when extracting features using LBP. Similar cases appear in SR, SSR, and HE.

**Table 2:** EER values and their improved ratio achieved using different feature extraction algorithms on UTFVP.

Data	EER Values/Improved Ratios		
	DWT [28]	LBP [29]	LPQ [30]
Original image	12.09%	8.62%	8.06%
IN	9.16%/24.2%	11.26%/−30.6%	5.83%/27.7%
SR	12.52%/−3.6%	7.5%/13.0%	7.78%/3.5%
SSR	14.71%/−21.7%	8.32%/3.5%	8.75%/−8.6%
HE	14.32%/18.4	8.65%/−0.3%	10.01%/−24.2
Proposed GFSSR	**8.88%/26.6%**	**5.70%/33.9%**	**4.31%/46.5%**

## 5. Conclusions

In this paper, an intensity variation normalization method was proposed for finger vein recognition. The method was based on a model that reasonably describes the effect of finger tissues on finger vein images. In this model, a captured finger vein image was regarded as the multiplication of the intensity variation and the reflectance images. To accurately obtain the intensity variation image, the guided filter was adopted since it has adaptive edge-preserving smoothing ability especially in local regions. The intensity variation component could then be normalized in the logarithm domain. The comparative experiments on image enhancement and matching accuracy demonstrated that the proposed method had better performance compared to the common methods for finger vein image enhancement and recognition. Taking into account that the capacities and percentages of the tissue components vary among a person, the adaptive current for each individual to capture high quality finger vein images will be investigated in our future research.
